# Phenyllactic Acid from *Lactobacillus plantarum* Promotes Adipogenic Activity in 3T3-L1 Adipocyte via Up-Regulation of PPAR-γ2

**DOI:** 10.3390/molecules200815359

**Published:** 2015-08-24

**Authors:** Soundharrajan Ilavenil, Da Hye Kim, Mariadhas Valan Arasu, Srisesharam Srigopalram, Ravikumar Sivanesan, Ki Choon Choi

**Affiliations:** 1 Grassland and Forage Division, National Institute of Animal Science, RDA, Seonghwan-Eup, Cheonan-Si, Chungnam 330801, Korea; E-Mails: ilavenil@korea.kr (S.I.); srigopalram82@korea.kr (S.S.); 2 The United Graduate School of Agricultural Sciences, Tottori University, Tottori-Shi 6808553, Japan; E-Mail: pioioiq@nate.com; 3 Department of Botany and Microbiology, Addiriyah Chair for Environmental Studies, College of Science, King Saud University, P. O. Box 2455, Riyadh 11451, Saudi Arabia; E-Mail: mvalanarasu@gmail.com; 4 Department of Biotechnology, PRIST University, Tamilnadu 613403, India; E-Mail: drravinikesh@yahoo.co.in

**Keywords:** phenyllactic acid, 3T3-L1 pre-adipocyte, adipocyte differentiation, troglitazone, PPAR-γ antagonist, glucose uptake, type-2 diabetes

## Abstract

Synthetic drugs are commonly used to cure various human ailments at present. However, the uses of synthetic drugs are strictly regulated because of their adverse effects. Thus, naturally occurring molecules may be more suitable for curing disease without unfavorable effects. Therefore, we investigated phenyllactic acid (PLA) from *Lactobacillus plantarum* with respect to its effects on adipogenic genes and their protein expression in 3T3-L1 pre-adipocytes by qPCR and western blot techniques. PLA enhanced differentiation and lipid accumulation in 3T3-L1 cells at the concentrations of 25, 50, and 100 μM. Maximum differentiation and lipid accumulation were observed at a concentration of 100 μM of PLA, as compared with control adipocytes (*p* < 0.05). The mRNA and protein expression of PPAR-γ2, C/EBP-α, adiponectin, fatty acid synthase (FAS), and SREBP-1 were increased by PLA treatment as compared with control adipocytes (*p* < 0.05). PLA stimulates PPAR-γ mRNA expression in a concentration dependent manner, but this expression was lesser than agonist (2.83 ± 0.014 fold) of PPAR-γ2. Moreover, PLA supplementation enhances glucose uptake in 3T3-L1 pre-adipocytes (11.81 ± 0.17 mM) compared to control adipocytes, but this glucose uptake was lesser than that induced by troglitazone (13.75 ± 0.95 mM) and insulin treatment (15.49 ± 0.20 mM). Hence, we conclude that PLA treatment enhances adipocyte differentiation and glucose uptake via activation of PPAR-γ2, and PLA may thus be the potential candidate for preventing Type 2 Diabetes Mellitus (T2DM).

## 1. Introduction

Adipose tissue is a metabolic and endocrine organ that plays an important role in the regulation of energy homeostasis. Adipocytes are potential therapeutic target for obesity, type-2 diabetes (T2DM) related diseases or disorder. Adipogenesis is a sequential process accompanied by coordinated changes in morphology, hormone sensitivity, and gene expressions. Peroxisome proliferator activated receptor-2 (PPAR-γ2), CCAAT/enhancer-binding protein-α (C/EBP-α), sterol regulatory element binding protein-1 (SREBP-1) adipocyte binding protein-2 (aP2), and FAS play important roles in regulation of adipogenesis [1].

Many studies have reported that a decrease in adipogenesis and its related gene expression are associated with T2DM [2]. Type-2 diabetes is characterized by insulin resistance and impaired insulin secretion. Insulin resistance is a condition in which muscle and adipose tissues lose their glucose uptake ability. This condition stimulates the secretion of higher amounts of insulin from pancreases. It causes high plasma insulin and glucose level, which leads to type-2 diabetes (T2DM) [3]. When adipose tissue fails to store the excessive energy as a triglyceride and it accumulated in the other than adipose tissue is called as ectopic fat, which may cause insulin resistance and insufficient insulin secretion in the pancreas [4]. Irregular adipogenesis leads to increases in plasma free fatty acid levels, which are normally stored in liver and muscle; this can stimulate insulin resistance and promote T2DM [5]. Adiponectin plays an important role in the regulation of glucose and lipid metabolism. In differentiated adipocyte, adiponectin over-expressing cells exhibited more fat accumulation, and it stimulate glucose uptake through activation of glucose transporter-4 (GLUT-4). PPAR-γ2 well know key transcriptional factor for activating many adipocyte-specific genes, and it is required for adiponectin expression [6].

Phenyllactic acid (PLA) is found in *Lactobacillus* spp. and produced during phenylalanine metabolism. These bacteria are commonly used in dairy, meat, and plant fermentation and also in food products like yogurt, cheese, kimchi, sauerkraut, sourdough, and pickles and it possessing potent antifungal, antioxidant and probiotic activities [7,8]. These bacteria provide certain tastes and flavors depending on the balance between volatile and non-volatile organic acids. PLA possesses a broad spectrum of antibacterial and antifungal activity [9]. Nowadays, a number of synthetic compounds are available in the market. These compounds are used as adipogenesis regulators, however, they have a number of adverse effects, therefore, isolation of new adipogenic regulator from natural sources plays an essential role in developing new therapeutic agents. For that purpose, we isolated and characterized PLA from *Lactobacillus* spp. [10] and planned to evaluate whether PLA can exert modulatory effects on adipocyte differentiation and lipid accumulation in 3T3-L1 pre-adipocytes.

## 2. Results and Discussion

### 2.1. 3T3-L1 Preadipocytes Proliferation Activity of PLA

**Figure 1:**
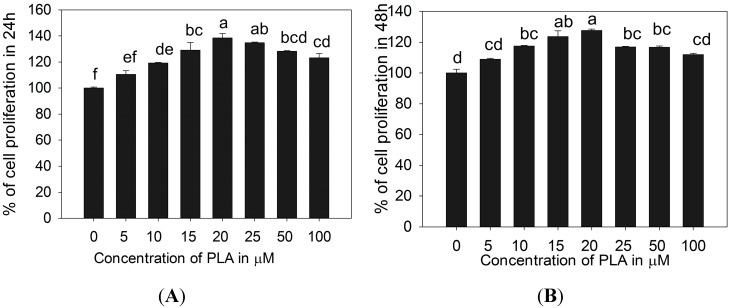
Cell proliferation activity of PLA. It increased cell proliferation in a concentration-dependent manner (5–20 μM) at 24 (**A**) and 48 h (**B**). Further increasing PLA from 25 to 100 μM reduced the % of cell proliferation, as compared with the previous concentration. The results represent the mean ± SEM of six replicates. Different letters a, b, c, d, e, f within a treatment indicate significant differences (*p* < 0.05).

Cell proliferation activity of PLA on 3T3-L1 pre-adipocytes was investigated using EZ-Cytox kit. PLA treatments (5, 10, 15, 20, 25, 50, and 100 µM) slightly influenced positive cell proliferation up to 20 µM. The further increment of PLA (25–100 µM) slightly reduced the cell proliferation after 24 and 48 h as compared with the previous dose of PLA. However, cells treated with PLA (25–100 µM) showed slight increases in cell proliferation compared with control (Figure 1).

### 2.2. Effect of PLA on Lipid Accumulation and Glycerol Release

**Figure 2:**
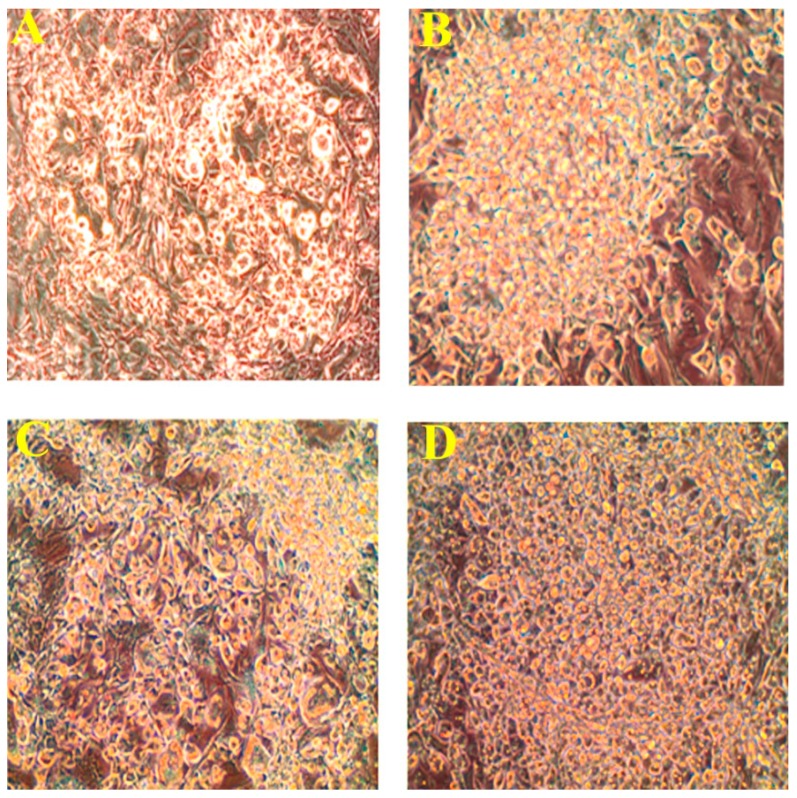
Microscopic (magnifications ×20) view of adipocyte differentiation on the 10th day. (**A**) Control; (**B**) 25 µM; (**C**) 50 µM; (**D**) 100 µM. Below 25 µM did not influence adipocyte differentiation.

Further, we investigated the effect of different concentration of PLA (5, 10, 15, 20, 25, 50 and 100 µM) on adipocyte differentiation. We found that the adipocyte differentiation was accelerated by PLA supplement at the concentration of 25–100 µM. The previous concentration of (5–20 µM) PLA did not influence adipocyte differentiation as compared to control cells. Adipocyte differentiation increased after 25 µM of PLA treatment compared with the control cells (Figure 2).

**Figure 3:**
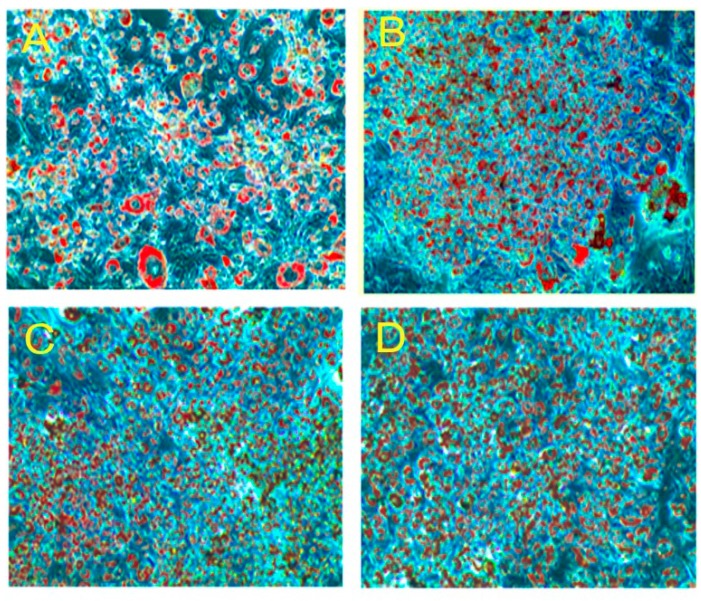

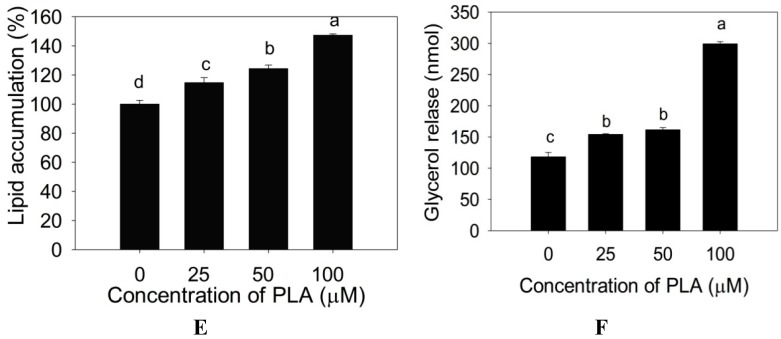
Oil Red O staining of lipid accumulation and glycerol release in adipocyte on the 10th day. (**A**) Control; (**B**) 25 µM; (**C**) 50 µM; (**D**) 100 µM; (**E**) % of lipid extracted from experimental adipocyte with 100% isopropyl alcohol; (**F**) Glycerol release from differentiated adipocytes in presence of different concentration of PLA. The results represent the mean ± SEM of six replicates. Different letters a, b, c, d within treatment indicate significant differences (*p* < 0.05).

Figure 3A–D lipid accumulation and glycerol release in the 3T3-L1 pre-adipocytes. PLA treated adipocytes exhibited a higher number of lipid droplets than the control. The percentage of lipid accumulation was greater in the adipocyte treated with different concentration of PLA when compared with control cells (*p* < 0.05) (Figure 3E). Glycerol release increased remarkably in differentiated adipocytes in the presence of PLA (Figure 3F). Hence, the PLA concentration at 100 µM showed the most efficient effect on adipocyte differentiation and lipid accumulation.

### 2.3. Quantification of Adipogenic mRNA and Their Proteins by qPCR and Western Blot

**Figure 4:**
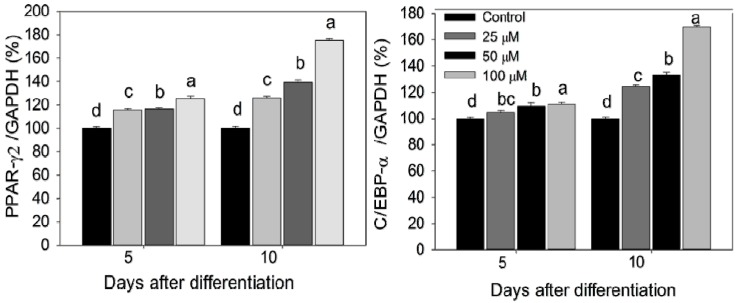

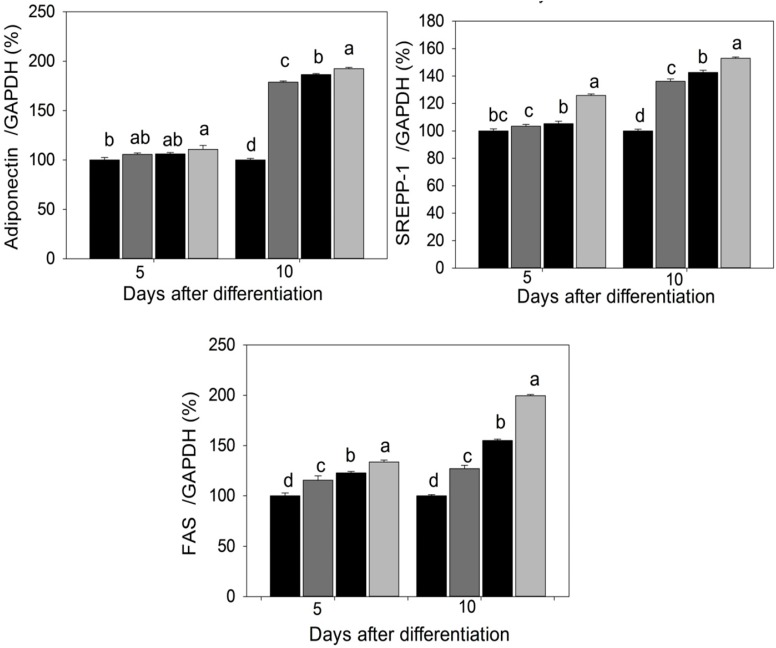
PPAR-γ2, C/EBP-α, adiponectin, SREBP-1 and FAS mRNA expression pattern in differentiated adipocytes were analyzed by qPCR. Data were shown as means ± SEM of six replicates. Different letters a, b, c, d within a treatment indicate a significant difference (*p* < 0.05).

**Figure 5:**
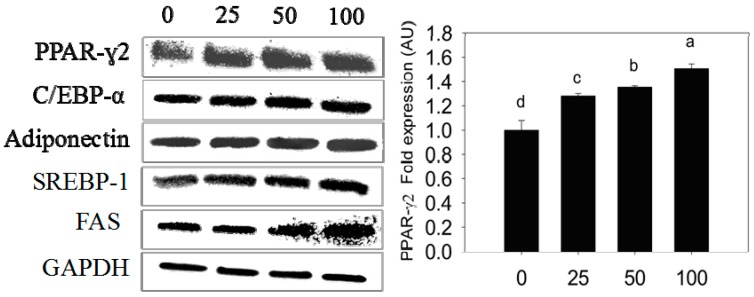

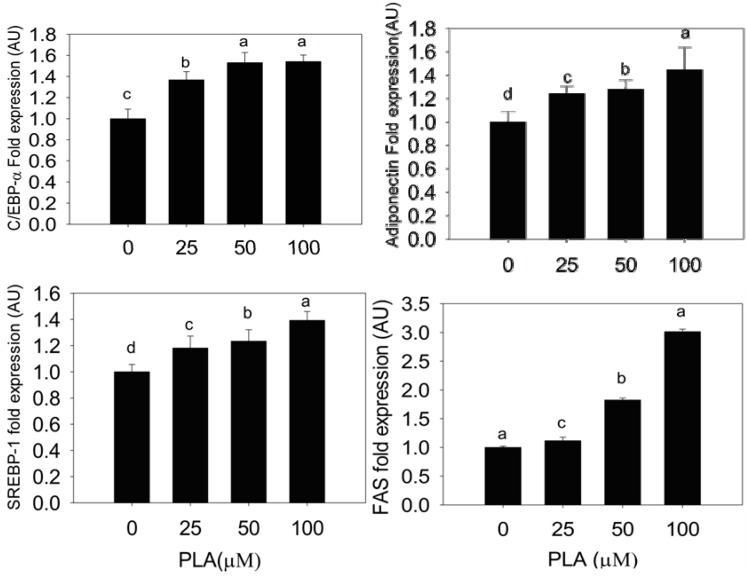
Immunoblot analyses of PPAR-γ2, C/EBP-α, adiponectin, SREBP-1, and FAS. Bar diagram indicates fold expression of proteins (arbitrary units-AU) in differentiated adipocytes quantified by the Imagej software. Data were shown as means ± SEM of three replicates. Different letters a, b, c, d within a treatment indicate a significant difference (*p* < 0.05).

The PPARγ2, C/EBP-α, adiponectin, FAS, and SREBP-1 mRNA and their protein expressions were investigated in control and experimental adipocytes by qPCR and western blot techniques. The 3T3-L1 pre-adipocytes treated with different concentrations of PLA accelerates the expression rate of PPARγ2 and C/EBP-α mRNA and their proteins compared with control cells. Subsequently, the expression of adiponectin, FAS, and SREBP-1 were stimulated in a dose-dependent manner. Maximum mRNA and their protein expressions were observed at 100 µM of PLA (Figure 4 and Figure 5).

**Figure 6:**
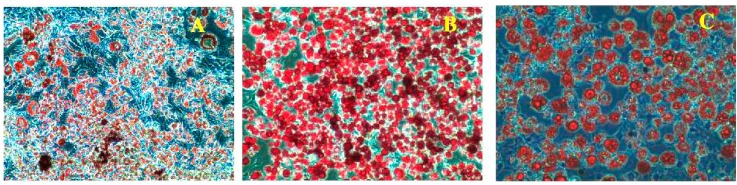

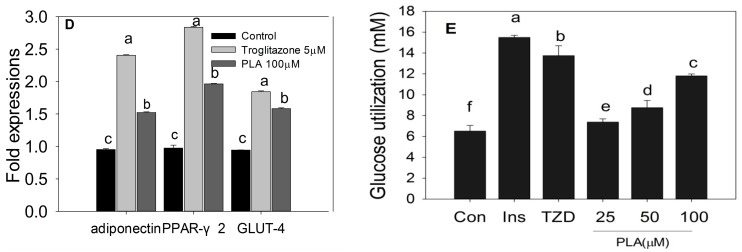
Effect of troglitazone and PLA on lipid accumulation and glucose utilization. (**A**) Lipid accumulation in control; (**B**) Lipid accumulation in troglitazone-treated cells (5 μM); (**C**) Lipid accumulation in the PLA-treated cell (100 μM); (**D**) adipocyte treated with troglitazone (5 μM) and PLA (100 μM) increased adiponectin, PPAR-γ2 and GLUT-4 expression in adipocytes; (**E**) Glucose utilization in 3T3-L1 cells by troglitazone (TZD), Ins- insulin, and different concentration of PLA. Data were shown as means ± SEM of six replicates. Different letters a, b, c, d, e, f within treatments indicate significant differences (*p* < 0.05).

The 3T3-L1 pre-adipocytes treated with troglitazone as an agonist for PPAR-γ2, (5 µM) for 48 h showed significantly increased adiponectin, PPAR-γ2, and GLUT-4 mRNA expression, compared with control cells. Similarly, we found increased expression of PPAR-γ2, adiponectin, and GLUT-4 mRNA in PLA-treated adipocytes, but these expressions were lower than with troglitazone treatment. Furthermore, PLA significantly enhanced glucose uptake in a concentration-dependent manner, these results are comparable with insulin and troglitazone treatments (Figure 6).

### 2.4. Discussion

Adipogenesis is driven by a complex transcriptional cascade pathway involving the continuous activation of PPAR-γ2 and C/EBP-α. C/EBP-α and C/EBP-γ were rapidly expressed after the hormonal induction of differentiation. These factors act synergistically to stimulate the PPAR-γ2 and C/EBP-α expression. This is a master adipogenic transcriptional factor. PPAR-γ2 and C/EBP-α together stimulate the adipocyte differentiation via activating the transcription of the genes that involved in creating and maintaining adipocyte phenotype. Many studies reported that the PPAR-γ is necessary as well as sufficient to promote adipogenesis, and the C/EBP-α is influenced by the maintaining the expression of PPAR-γ2 [11]. The present study, supplement of PLA to the 3T3-L1 preadipocytes during the adipocyte differentiation increases expression of PPAR-γ2 and C/EBP-α mRNA and their protein level. These data suggest that PLA stimulate the adipogenesis via up- regulation of PPAR-γ2 and C/EBP-α.

Adiponectin is an adipocyte-derived hormone that is expressed in differentiated adipocytes. It stimulates lipid accumulation and insulin-responsive transporter [12]. Over-expression of adiponectin in stably transduced 3T3-L1 adipocyte that stimulates adipocyte differentiation by its autocrine effects [13]. Our study indicated that the PLA enhances the adiponectin expression in differentiated adipocytes. It indicated that the PLA strongly induce the adipocyte differentiation by adiponectin secretion. Adiponectin gene transcription is induced by PPAR-γ through a PPRE in its promoter [14] and by C/EBP through an intronic enhancer [15]. Here we found that PLA enhances adiponectin in differentiated adipocytes on the 5th and 10th day might be due to PPAR-γ2 and C/EBP expression. Adiponectin promotes cell proliferation and differentiation in 3T3-L1 pre-adipocyte and augments programmed gene expression, which is responsible for adipogenesis and increasing cytoplasmic accumulation and insulin responsiveness of the glucose transport system in the adipocyte [13]. Increases of adiponectin secretion would have more favorable effects against insulin resistance as well as the atherogenic and inflammatory process [16,17]. Based on these results, pre-adipocyte exposed to PLA not only increased lipid droplet and glycerol accumulation, but also increased adiponectin mRNA, protein level and glucose utilization during differentiation.

The expression of SREBP-1 is increased in liver, white and brown adipocytes. However, higher expression of SREBP-1 is found in adipocytes as compared with other fibroblasts. It is expressed during the adipocyte differentiation [18]. SREBP-1 enhances other factors such as FAS, and acetyl carboxylase [17], glycerol-3-phosphate acyltransferase [19] and lipoprotein lipase [20]. Further, it promotes synthesis of natural fatty acid through fatty acid metabolism. SREBP-1 regulates adipocyte differentiation under the control of the PPARγ2 transcription factor [21]. Our study, SREBP-1 mRNA, and their protein expression were increased in differentiated adipocytes after differentiation induction. Further, this SREBP-1 expression was accelerated by different concentration of PLA treatments. This data suggest that the PLA significantly activate the adipocyte differentiation by activating SREBP-1 through PPARγ2 stimulation. The ADD-1/SREBP-1c plays an important role in the regulation of adipogenic gene expression by up-regulating lipogenesis and down-regulating fatty acid oxidation [22,23,24].

Fatty acid synthase (FAS) is an important enzyme in fatty acid metabolism, and it catalyzes the *de novo* synthesis of long-chain fatty acids from acetyl CoA and malonyl CoA via an NADPH-dependent reaction [25]. ADD-1/SREBP can stimulate FAS, and lipoprotein lipase, which are key regulators of fatty acid metabolism. Fat cells have the ability to synthesize triglycerides from fatty acids, which are provided by circulating lipoproteins or through endogenous fatty acid biosynthesis [26]. The ADD-1/SREBP regulates fatty acid metabolism via PPARγ2 activation and also induces FAS and S14 gene promoters [18,27,28]. According to qPCR and western blot results, the increases of FAS mRNA and their protein expression were associated with PLA induced lipid accumulation in adipocytes. From that we suggest that PLA stimulates adipogenesis by activating adipogenic specific genes such as PPAR-γ and C/EBP-α, plays a key regulatory role.

Thiazolidinediones (TZDs) as activators of PPAR-γ2 are the first line of agents that directly target the adipocytes. TZDs improve the insulin sensitivity by decreasing peripheral insulin resistance and thus lower blood glucose level in type-2 diabetes patients. TZDs stimulate adipocyte differentiation via increasing the number of the small adipocytes. Small adipocytes are more sensitive to insulin than large adipocytes [29,30]. PPAR-γ activation leads to stimulated glucose uptake, storage of triglycerides and production of adiponectin. It also induces increased GLUT-4 mRNA levels in adipocytes [31]. Muscle and adipose tissue contain at least two kinds of glucose transporter, GLUT-1 and GLUT-4, among which GLUT-4 is an important insulin regulator [32]. Insulin induces glucose uptake in adipocytes by binding to its receptor proteins within cells, leading to the translocation of GLUT4 to the cell surface [33]. In our results, 3T3-L1 pre-adipocytes exposed to troglitazone showed increased adiponectin, PPAR-γ2, and GLUT-4 expression during differentiation and enhanced glucose uptake in adipocytes. PLA-treated adipocytes also exhibited increased adiponectin, PPAR-γ2, GLUT-4 mRNA expression and glucose uptake. Adiponectin increases insulin sensitivity by stimulating the fatty acid oxidation, reduces triglycerides, and improves the glucose metabolism [34]. Taken together, the statements above suggest that PLA-enhanced glucose uptake in cells may be due to up-regulation of adiponectin, PPAR-γ2, and GLUT-4 mRNA levels.

## 3. Experimental Section

### 3.1. Chemicals

The mouse 3T3-L1 pre-adipocyte cell line was obtained from ATCC (CL-173, Manassas, VA, USA). The fetal bovine serum (FBS) and Dulbecco’s modified Eagle’s medium (DMEM) were from Gibco-BRL (Grand Island, NY, USA). The mRNA extraction and RT-PCR kits were from Invitrogen (Carlsbad, CA, USA). Troglitazone was from Sigma Aldrich (St. Louis, MO, USA) and T0070907, a PPAR-γ2 antagonist, was from Selleckchem (Houston, TX, USA). Primers were from Bioneer Corp. (Daejeon, Korea) and other chemicals were from Sigma (St. Louis, MO, USA) or SPL Life Sciences (Pochun, Korea).

### 3.2. Isolation and Characterization of PLA from Lactobacillus spp. KCC-10

Fresh *Lactobacillus* spp. KCC-10 was cultured in a 5 L Erlenmeyer flask containing 3 L MRS broth with 2% glucose for 3 days at 30 °C. At the end of the fermentation cycle, the supernatant was collected by centrifugation at 10,000 *g* for 30 min. The supernatant was extracted with ethyl acetate (3 × 300 mL). The solvent phase was concentrated using a vacuum at 35 °C to obtain the crude extract. The ethyl acetate extract was purified with a combined hexane/ethyl acetate solvent system. The isolated compound was subjected into FTIR, ^1^H-NMR (300 MHz) and ^13^C-NMR (75.45 MHz) for identification and characterization of the isolated compound. These data were already published [10].

### 3.3. Cell Proliferation

The water-soluble tetrazolium salt WST [2[2-methoxy-4-nitrophenyl]-3[4-nitrophenyl]-5-[2,4-disulfophenyl]-2-*H*-tetrazolium monosodium salt] was used for analysis of the viability of 3T3-L1 pre-adipocytes. The cells were seeded in the 96 well at a density of 1 × 10^4^ cells/well. The cells were exposed to the different concentration of PLA. It was incubated at the 37 °C in 5% CO_2_ incubator for 24 and 48 h, and then the culture was treated with WST incubated for 2 h. Then, the intensity of colour was measured at 450 nm using spectra count ELISA reader (Packard Instrument Co., Downers Grove, IL, USA).

### 3.4. Differentiation

Adipocyte differentiation was induced according to the method of Choi *et al.* with modifications [35]. Briefly, 3T3-L1 pre-adipocytes were seeded in 12 well culture plats at a density of 2 × 10^4^ cells/well. Cells were incubated at 37 °C with 5% CO_2,_ and culture medium was replaced by every 48 h. After two days of 100% confluence of 3T3-L1 cells, the growth media was replaced by differentiation media (DMEM containing 10% FBS, 0.5 mM 3-isobutyl-1-methylxanthine, 1 μM dexamethosone, and 1.7 μM insulin) for 48 h. The pre-adipocytes were maintained and re-fed every day with 10% FBS-DMEM medium. To examine the effect of PLA on adipocytes differentiation, the pre-adipocytes received 5, 10, 15, 20, 25, 50 and 100 µM) every 2 days started at 2 days post confluence until the end of the experiment days. Troglitazone used as an agonist for PPAR-γ2.

### 3.5. Oil Red O Staining

Differentiated adipocytes in the presence of PLA were fixed in 10% formalin for 1 h and then washed twice with 60% isopropanol. The fixed cells were stained with 0.35% oil Red O for 10 min at 37 °C and washed twice with *d*-H_2_O [36]. Photographs were taken with an inverted microscope (CKX41, Olympus Corp., Tokyo, Japan). The oil red O stain was extracted from adipocytes with 100% isopropanol and read at 490 nm.

### 3.6. Measurement of Glycerol Accumulation

According to adipolysis assay Kit procedure (EMD Millipore Corporation, Billerica, MA, USA), the glycerol level was quantified. In detail, differentiated adipocytes in the presence of different concentration of PLA (25, 50 and 100 μM) were washed twice with PBS and further incubated with lipolysis buffer for 1 h. Then, the buffer was collected and measured the glycerol concentration using microplate reader at 540 nm. The amount of glycerol was calculated using a standard curve of glycerol.

### 3.7. Glucose Utilization Test

The glucose uptake assay was conducted by the method of Vishwanath *et al.* with slight modifications [37]. The differentiated adipocytes were exposed to high glucose (25 mM) containing DMEM medium with PLA (25, 50 and 100 μM), insulin (1 μg/mL) and troglitazone (5 μM), individually for 24 h. An assay, without PLA was considered as control (0). After 24 h, the concentration of glucose in spent medium was estimated with a commercial assay kit (Sigma Aldrich-GAGO-20).

### 3.8. Quantification of PPRA-γ, C/EBP-α, Adiponectin, SREBP-1, FAS, and GLUT-4 mRNA Expression

Total RNA was extracted and quantified with the RNeasy lipid tissue mini kit (Qiagen, Germantown, MD, USA) and UVS-99. One microgram of RNA was reverse-transcribed using oligo(dT) and reverse transcriptase (Superscript III first-stand synthesis system for RT-PCR, Invitrogen). Real-time PCR was carried out with an ABI 7500 Real-Time PCR System. Target gene expression level was determined by SYBR green-based real-time PCR in 10-µL reactions containing 5 µL Power SYBR Green Master Mix (Applied Biosystems, Foster City, CA, USA), 1 µL cDNA, 1 µL 10 pmole forward (FP) and reverse primers (RP) and 3 µL DEPC water. Primer details (PPRAγ2-FP: gtgctccagaagatgacagac, RP: ggtgggactttcctgctaa, C/EBP-α-FP: gcaggaggaagatacaggaag, RP: acagactcaaatcccaaca, adiponectin-FP: ccgttctcttcacctacgac, RP: tccccatccccatacac, FAS-FP: cccagcccataagagttaca, RP: atcgggaagtcagcacaa, SREBP-1-FP: gaagtggtggagagacgcttac (F) RP: tatcctcaaagggctggactg, GLUT-4 FP: cccacagaaggtgattgaac, RP: gagagagcccagagcgtag and G3PDH-FP: cggtgctgagtatgtctgtg, RP: ggtggagatgatgacccttt).

### 3.9. Western Blot

Proteins were extracted from differentiated adipocytes using RIPA lysis buffer with 1× protease inhibitor cocktail (Roche, Basel, Switzerland). The samples were centrifuged at 8000 *g* for 10 min at 4 °C. The total protein concentration was measured by a Bio-Rad protein assay kit. An equal amount of protein samples were separated by SDS-PAGE (10%) and transblotted onto polyvinylidene difluoride (PVDF) membranes (iBlot gel transfer stacks, Novex, Life Technology, Waltham, MA, USA). According to western breeze chemilumeniscence protocol (Invitrogen), the immunobloting was performed (except primary antibody incubation time and temperature) with rabbit monoclonal antibody of specific proteins such as PPRAγ2, C/EBP-α, and adiponectin, FAS, SREBP-1 and GAPDH. (Cell Signaling Technology, Danvers, MA, USA). The signals were observed with an enhanced chemilumeniscence kit (Bio-Rad-USA) by a chemiluminescence imaging system (Davinch Chemi, Seoul, Korea) and the intensity of immune reacted bands was quantified by the ImageJ software (1.49 version(32 bit), Wayne Rasband, National Institute of Health, Bethesda, MD, USA).

### 3.10. Statistical Analysis

Data were obtained from six replicates and analyses were carried out with MS Excel and the SPSS software (ver. 16; SPSS Inc., Chicago, IL, USA). Results were presented as means ± standard error. Differences between the means were analyzed using multivariate analysis with a significant level of *p* < 0.05.

## 4. Conclusions

Our data revealed that PLA supplementation accelerates adipocyte differentiation and lipid accumulation in 3T3-L1 pre-adipocytes through activation of PPAR-γ2 and the associated genes responsible for adipogenesis. Further, it stimulates the glucose uptake in the 3T3-L1 pre-adipocytes. Therefore, we suggested that the present form of the identified molecule may be a potential candidate for preventing T2DM.
